# Familial Parkinson's Disease Mutant E46K **α**-Synuclein Localizes to Membranous Structures, Forms Aggregates, and Induces Toxicity in Yeast Models

**DOI:** 10.5402/2011/521847

**Published:** 2011-07-09

**Authors:** Michael Fiske, Michael White, Stephanie Valtierra, Sara Herrera, Keith Solvang, Alina Konnikova, Shubhik DebBurman

**Affiliations:** Biology Department, Lake Forest College, Box P7, 555 North Sheridan Road, Lake Forest, IL 60045, USA

## Abstract

In Parkinson's disease (PD), midbrain dopaminergic neuronal death is linked to the accumulation of aggregated **α**-synuclein. The familial PD mutant form of **α**-synuclein, E46K, has not been thoroughly evaluated yet in an organismal model system. Here, we report that E46K resembled wild-type (WT) **α**-synuclein in *Saccharomyces cerevisiae* in that it predominantly localized to the plasma membrane, and it did not induce significant toxicity or accumulation. In contrast, in *Schizosaccharomyces pombe*, E46K did not associate with the plasma membrane. Instead, in one strain, it extensively aggregated in the cytoplasm and was as toxic as WT. Remarkably, in another strain, E46K extensively associated with the endomembrane system and was more toxic than WT. Our studies recapitulate and extend aggregation and phospholipid membrane association properties of E46K previously observed *in vitro* and cell culture. Furthermore, it supports the notion that E46K generates toxicity partly due to increased association with endomembrane systems within cells.

## 1. Introduction

Parkinson's disease (PD) is a fatal and incurable neurodegenerative disorder characterized by resting tremors, postural instability, and bradykinesia [1]. The death of midbrain substantia nigra neurons characterizes the pathology of both familial and sporadic forms of PD. Two types of molecules accumulate within these dying cells: misfolded and aggregated *α*-synuclein in cytoplasmic inclusions called Lewy bodies [2] and oxidative free radicals [1, 3]. Therefore, a major hypothesis is that *α*-synuclein misfolding and aggregation is linked to neuronal toxicity in PD [4–6]. While the molecular basis of sporadic PD is still unclear, mutations in six genes cause familial PD [7], including three point mutations (A53T, A30P, and E46K) within the *α*-synuclein gene [8–10]. 

In addition to self-aggregation [11–13], several other *α*-synuclein properties that may be relevant to PD include maintenance of neurotransmitter vesicular pools [14, 15], synaptic plasticity [16, 17], phospholipid binding and lipid metabolism [18–21], microtubule binding [22], chaperone-like ability [23–25], and ER-Golgi trafficking [26, 27]. While the A30P and A53T familial mutants are widely studied in how they alter the above properties to cause cytotoxicity and contribute to PD pathogenesis [28], less is known about the third and most recently discovered familial mutant, E46K. 

Thus far, E46K is known to aggregate into fibrils more rapidly than wild-type *α*-synuclein *in vitro* [29, 30] and in cell culture [31]. Additionally, recent biochemical studies suggest that E46K enhances contact between the amino and carboxyl domains although whether this interaction increases the likelihood of aggregation is still being sorted [32]. In contrast to the A30P and A53T familial mutants, E46K exhibits a reduced accumulation of early oligomers [13, 33, 34]. Like wild-type *α*-synuclein, E46K can form ion channel-like pores in lipid bilayers [34] although recent work suggests that these channels are less conductive than those formed by wild-type *α*-synuclein [35]. On the other hand, E46K binds phospholipids more readily than wild-type *α*-synuclein [29, 36, 37] and is more potent in inducing toxicity in neuroblastoma cells that are cotreated with cytokines [38]. In living cells, however, little is still known of E46K's ability to bind either the plasma membrane or internal membrane systems. Nor is it clear how E46K aggregation and/or membrane association might cause cellular toxicity.

Budding and fission yeast models have advanced our molecular understanding of several neurodegenerative diseases, including PD [21, 39–42]. Yeasts revealed that *α*-synuclein binds plasma membranes extensively in addition to its propensity to aggregate, but the relative importance of aggregation and membrane binding to toxicity is still unresolved [21, 43–46]. Yeasts have also provided evidence that conditions for *α*-synuclein toxicity can be varied. Some models demonstrate *α*-synuclein expression and dose-dependent aggregation itself causes toxicity [21, 44], while others report that *α*-synuclein-dependent toxicity, especially when moderately expressed, requires additional factors, such as proteasomal inhibition and oxidative stress [43, 45], or tau coexpression [43]. In 2003, Willingham et al. identified eighty-six yeast genes that were synthetically lethal with *α*-synuclein [47]. Similarly, disruption of the secretory pathway [44] and ER-Golgi trafficking [26, 27] were first found in yeast to increase *α*-synuclein toxicity. 

Here, we exploited the utility of both budding yeast and fission yeasts to test the hypothesis that the E46K mutant aggregates and binds membranes more strongly than WT *α*-synuclein in living organisms and that it accumulates to induce cellular toxicity. Indeed, we found that E46K binds the plasma membrane in budding yeast and associates with endomembrane systems and form intracellular aggregations in fission yeast. While E46K is not toxic to budding yeast, it is toxic to fission yeast especially when associated with internal membrane systems. This E46K-enhanced toxicity is best correlated with reduced cellular growth.

## 2. Results

As described in our past studies [45, 46], we tagged *α*-synuclein with GFP at its C-terminus and induced its expression with galactose in budding yeast and by removal of thiamine (the repressor) in fission yeast.

### 2.1. E46K Is Selectively Toxic in Fission Yeast but Not in Budding Yeast

Our first goal was to assess whether E46K was toxic to budding and fission yeasts, and we report that E46K is toxic to yeasts, albeit in a species- and strain-specific manner.

**Figure 1:**
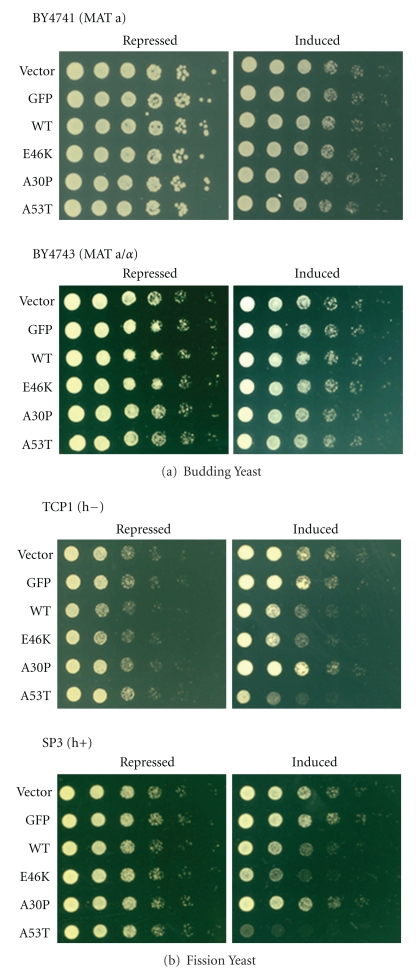
E46K exhibits species- and strain-specific toxicity in yeasts. (a) LEFT—BY4741 (mat a) and BY4743 (diploid mat a/*α*) budding yeast cells expressing WT, E46K, A30P, or A53T *α*-synuclein were serially diluted 5-fold and spotted onto repressing (glucose) or inducing (galactose) plates and grown for two days. Vector alone and GFP served as controls. No E46K-dependent *α*-synuclein toxicity was observed in either strain. (b) LEFT—TCP1 and SP3 fission yeast cells expressing WT, E46K, A30P, or A53T *α*-synuclein were serially diluted 5-fold and spotted onto repressing (+thiamine) or inducing (−thiamine) plates. Similar to budding yeast, vector alone and GFP served as controls. In both strains, WT, E46K, and A53T were toxic, but not A30P. In SP3, E46K was more toxic than WT.

First, for budding yeast, we examined two haploid strains of opposite mating types (BY4741 mat a and BY4742 mat *α*) and a diploid strain (BY4743 mat a/*α*). E46K was not toxic to any strain, as assessed by two assays: serial dilution spotting on solid growth plates (Figure 1(a); BY4742 not shown) and growth curves from liquid culture (data not shown). BY4741 cells that expressed E46K grew equally well as cells that did not express *α*-synuclein (vector alone and GFP) and those that expressed WT *α*-synuclein or two other familial mutants (A30P and A53T; Figure 1(a), *top*). Therefore, as we had previously reported for WT, A30P, and A53T [45], E46K *α*-synuclein did not adversely affect budding yeast growth. Additionally, neither the mating type (data not shown) nor ploidy doubling (Figure 1(a), *bottom*) altered the lack of E46K *α*-synuclein-dependent toxicity in budding yeast.

Next, we examined how *α*-synuclein affected the growth of two haploid fission yeast strains of opposite mating types: h− (TCP1) and h+ (SP3). In contrast to our budding yeast findings, we found that several *α*-synuclein variants were toxic to fission yeast (Figure 1(b)). Firstly, when compared to vector alone and GFP expressing cells, WT, E46K and A53T *α*-synuclein expressing cells were each toxic in both strains, but not A30P (Figure 1(b)). In TCP1, E46K and WT were toxic to similar extents but less than A53T (Figure 1(b), *top*). However, the strength of E46K toxicity was strikingly strain-specific. In SP3, E46K was more toxic than WT yet still less than A53T (Figure 1(b), *bottom*).

### 2.2. E46K Localizes to the Plasma Membrane in Budding Yeast

**Figure 2:**
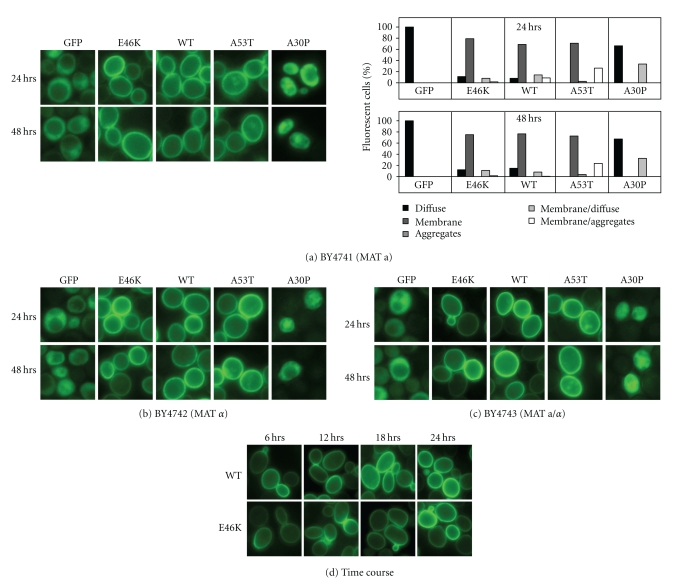
E46K associates with the plasma membrane in budding yeast. (a) Live cell GFP microscopy of BY4741 cells expressing WT, E46K, A30P, or A53T *α*-synuclein and GFP alone at 24 and 48 hours of expression (*left*).* Quantification:* ~750 cells of each type were scored for these localization patterns: cytoplasmically diffuse, plasma membrane, aggregate, plasma membrane/diffuse, and plasma membrane/aggregate (*right*). Phenotypes were plotted as a percent of total cells that fluoresced. (b) BY4742 and (c) BY4743 cells expressing WT, E46K, A30P, or A53T *α*-synuclein at 24 and 48 hours of expression. In each of these three strains, E46K consistently localized to the plasma membrane just like WT and A53T, while A30P and GFP alone were predominantly cytoplasmically diffuse.

To gain insight into E46K *α*-synuclein's differential toxicity in yeasts, we asked where it localized within the cells. Using live cell GFP imaging, we first tracked its localization in budding yeast. E46K *α*-synuclein consistently localized to the plasma membrane of BY4741 yeast during 48 hours of expression, similar to WT and A53T *α*-synuclein localization patterns (Figure 2(a)) that we previously had reported for BY4741 [45]. Quantification of localization in fluorescing cells indicates that more than 90% expressed E46K *α*-synuclein at the plasma membrane (Figure 2(a)). Unlike A53T, however, E46K *α*-synuclein was rarely seen aggregated in the cytoplasm (Figure 2(a)). Nor did we observe any E46K localization to internal endomembrane systems. In contrast, A30P *α*-synuclein was mostly cytoplasmically diffuse, similar to GFP alone (Figure 2(a)) [45].

E46K plasma membrane association and the patterns of localization of the other *α*-synuclein variants were maintained in BY4742 (Figure 2(b)) and BY4743 (Figure 2(c)) strains. We next wondered if E46K bound the plasma membrane more extensively than WT *α*-synuclein but found no difference in the extent of plasma membrane association between them over the first 24 hours of expression: both appeared at the plasma membrane as early as 6 hours to similar extents (Figure 2(d)). 

Thus, the lack of E46K *α*-synuclein toxicity in budding yeasts correlated with plasma membrane association.

### 2.3. E46K Aggregates and Associates with the Endomembrane System in Fission Yeast

**Figure 3:**
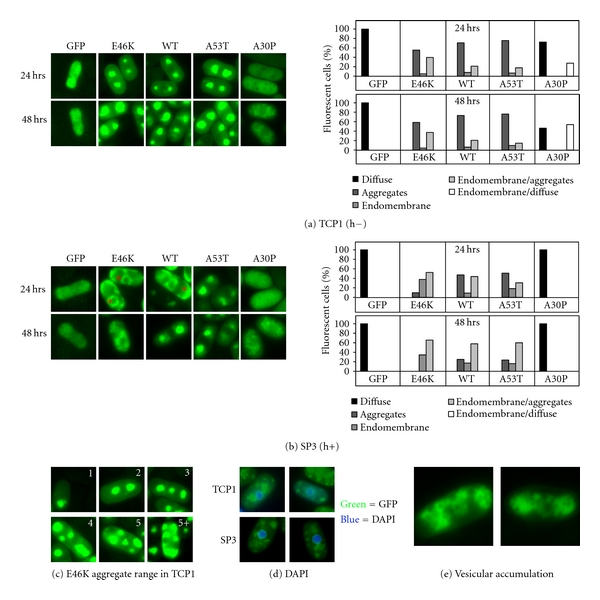
E46K *α*-synuclein exhibits strain-specific aggregation and endomembrane localization in fission yeast. (a and b) Live cell GFP microscopy of TCP1 cells (a) and SP3 cells (b) expressing WT, E46K, A30P, or A53T *α*-synuclein, and GFP alone, at 24 and 48 hours of expression (*left*). *Quantification:* ~750 cells of each type were scored for these localization patterns: cytoplasmically diffuse, aggregate, endomembrane, endomembrane/diffuse, endomembrane/aggregate (*right*). Phenotypes were plotted as a percent of total cells that fluoresced (*N* = 2). In TCP1, E46K *α*-synuclein mostly aggregated in TCP1, like WT and A53T. In SP3, E46K *α*-synuclein became prominenty localized to endomembrane systems, more so than WT and A53T. A30P was cytoplasmically diffuse like GFP in both strains. (c) E46K aggregates mostly varied from 1–5 per cell, but as many as 10 aggregates per cell were observed. (d) DAPI staining indicated that E46K localization in both strains (TCP1: top; SP3: bottom) was outside the nucleus. (e) E46K cells often exhibited prevacuolar localization and vesicular accumulation.

In stark contrast to budding yeast, E46K *α*-synuclein was prominently absent at the plasma membrane when examined in the two haploid fission yeast strains (Figure 3). In TCP1, E46K aggregated in the cytoplasm, again strikingly similar to WT and A53T *α*-synuclein (Figure 3(a)) that we previously had reported in TCP1 [46]. A30P *α*-synuclein and GFP alone were cytoplasmically diffuse (Figure 3(a)), also as previously reported [46]. Most cells had 1–3 aggregates, but some had up as many as 5–10 per cell (Figure 3(c)). These aggregates were outside the nucleus, as determined by DAPI staining (Figure 3(d), *top*). Notably, DAPI staining typically altered the pattern of *α*-synuclein localization, reducing the size and location of *α*-synuclein puncta, which suggests that some of the larger accumulations were either a collection of dispersible smaller aggregates or compact membranous vesicles (Figure 3(d), *top*).

However, in the second strain SP3, E46K localization was strikingly different. Initially, up to 24 hours of expression, both E46K and WT *α*-synuclein distributed to the membrane linings of many intracellular compartments, in addition to aggregating in the cytoplasm (Figure 3(b)). Over the next 24 hours, 76% WT *α*-synuclein expressing cells contained aggregates similar to TCP1, while E46K *α*-synuclein was even more prominently localized to diverse intracellular locations (98% cells), with no cells containing just aggregates (Figure 3(b)). Over this same course of time, A30P *α*-synuclein remained cytoplasmically diffuse while, A53T was mostly aggregated by 48 hours (84%), with endomembrane localization less extensive than E46K (16%; Figure 3(b)). This E46K intracellular localization pattern excluded the nucleus but not the nuclear envelope (Figure 3(d), *lower*), and prominently lit up perinuclear and vacuolar/prevacuolar compartments (Figure 3(b)), and other components of the endomembrane system that need further identification. Often, vesicular accumulations were observed within cells: mostly with E46K, less with WT, and rarely with A53T (Figure 3(e)).

Thus, the added toxicity of E46K *α*-synuclein in SP3 fission yeast appears correlated best with its localization to diverse endomembrane organelles. 

### 2.4. E46K Localization in Fission Yeast Is Concentration Dependent

**Figure 4:**
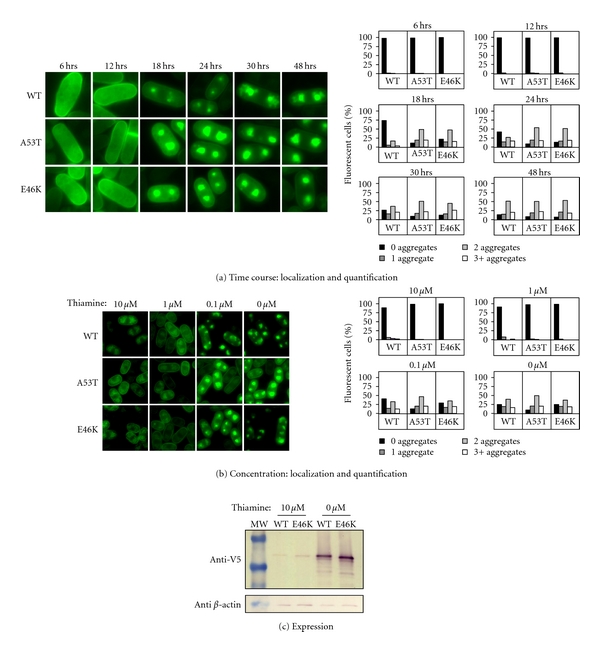
E46K *α*-synuclein aggregates in a time- and concentration-dependent manner in fission yeast. (a) Time course: *Left*—TCP1 cells expressing WT, A53T, or E46K *α*-synuclein (grown on media without thiamine) visualized using a fluorescent microscope over a 48-hour time period. *Right*—Quantification of the number of *α*-synuclein aggregates in each cell plotted as a percent of total cells that fluoresced (*N* = 2). E46K and A53T *α*-synuclein aggregated more intensely and quickly than WT. (b) Concentration: *Left*—TCP1 cells expressing WT, A53T, or E46K *α*-synuclein were grown in decreasing concentrations of thiamine (10, 1.0, 0.1, and 0.0 *μ*M) to induce increasing expression of *α*-synuclein and cells were visualized using a fluorescent microscope at 24 hours. *Right*—Quantification of number of aggregates in each cell plotted as a percent of total cells that fluoresced (*N* = 2). All three *α*-synuclein variants aggregated in a concentration-dependent manner but only at 0.1 and 0.0 *μ*M thiamine. *Middle—*Western blot of TCP1 cells expressing WT or E46K *α*-synuclein grown in 0 *μ*M or 10 *μ*M thiamine concentration indicates a low level of expression even in strongly repressing media (10 *μ*M thiamine).

We previously reported that WT and A53T *α*-synuclein aggregate in a time- and concentration-dependent manner in fission yeast, providing live cell support for the nucleation polymerization hypothesis [46]. Whether E46K does the same has not been reported. Indeed, we found that E46K *α*-synuclein localization in TCP1 was time-dependent (Figure 4(a)). Over a time course of 48 hours, E46K was initially diffuse in the cytoplasm for the first 12 hours, but thereafter aggregated into one or more punctate aggregates within individual cells (Figure 4(a)). By 18 hours, less than 5% cells had cytoplasmically diffuse E46K while, WT *α*-synuclein was cytoplasmically diffuse in 10% cells even at 30 hours (Figure 4(a)). 

Importantly, the E46K pattern of aggregation closely resembled A53T *α*-synuclein aggregation (Figure 4(a)). Remarkably, at no point over the time course was E46K *α*-synuclein (nor WT or A53T) localized at the plasma membrane (Figure 3(a)). A30P *α*-synuclein remained cytoplasmically diffuse and never aggregated over this same time course (data not shown). Similarly, E46K *α*-synuclein also aggregated in TCP1 in a concentration dependent manner, which was demonstrated by growing E46K cells in the presence of decreasing amounts of the repressor, thiamine (10, 1.0, 0.1, and 0 *μ*M; Figure 4(b), *left*). At the two highest thiamine concentrations, E46K *α*-synuclein was diffusely localized in the cytoplasm (Figure 4(b), *left*). However, at the lower thiamine concentrations as more protein is expressed, E46K started aggregating in most cells, similar to WT and A53T *α*-synuclein (Figure 4(b), *left*). The low level of *α*-synuclein observed by GFP fluorescence even at the highest thiamine concentration was confirmed by Western blotting (Figure 4(c), *right*).

Thus, E46K *α*-synuclein aggregated in a time- and concentration-dependent manner, supporting the nucleation polymerization model. Alternatively, the data can also be explained by concentration-dependent membrane localization as well, if the E46K puncta are, in fact, compact membrane structures, rather than aggregates.

### 2.5. E46K Modestly Accumulates in Fission Yeast and Is Phosphorylated in Both Yeasts

**Figure 5:**
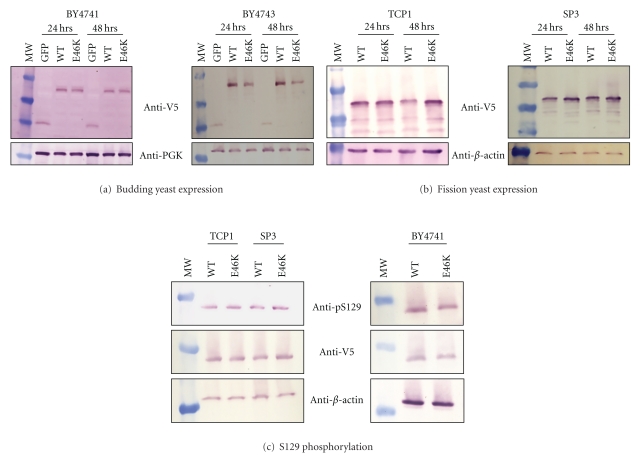
E46K *α*-synuclein expression in budding and fission yeasts. (a) Western blot of *α*-synuclein in budding yeast lysates of BY4741 (*left*) and BY4743 (*right*) at 24 and 48 hours of expression. E46K expression is similar to WT in BY4741 or lesser in BY4743. (b) Western blot of *α*-synuclein in fission yeast lysates of TCP1 (*left*) and SP3 (*right*) at 24 and 48 hours of expression. E46K expression is more than WT in TCP1 (24 hrs) and SP3 (24 and 48 hrs). (c) Western blot of budding yeast (BY4741) and fission yeast (TCP1 and SP3) expressing WT and E46K for 24 hours, probed with serine-129 phosphorylation-specific antibody (ab59264; Abcam), V5 antibody (for total *α*-synuclein), and *β*-actin (protein loading control). WT and E46K are phosphorylated at serine-129 in both yeasts.

We wondered if the strain-specific enhancement of E46K *α*-synuclein toxicity in fission yeast that was lacking in budding yeast was partly due to its selective accumulation in fission yeast. In budding yeast, E46K and WT *α*-synuclein were indeed either similar (BY4741) or even lower (BY4743) at 24 and 48 hours of expression, indicating a lack of accumulation (Figure 5(a)). In fission yeast, however, we did observe a slight increase in E46K *α*-synuclein expression relative to WT in both TCP1 (48 hrs) and SP3 strains (both 24 and 48 hrs; Figure 5(b)). Thus, E46K *α*-synuclein toxicity may be linked to this modest accumulation.

Given that serine phosphorylation is linked to *α*-synuclein related PD pathology [48–50], we next asked if E46K toxicity is linked to phosphorylation. Firstly, we found that both E46K and WT *α*-synuclein were phosphorylated at serine-129 in both budding and fission yeasts and to the same extent (Figure 5(c)). Importantly, the enhanced E46K toxicity in SP3 was not correlated with altered level of *α*-synuclein phosphorylation (Figure 5(c)). 

### 2.6. E46K Exhibits Reduced Growth and Survival in Fission Yeast

**Figure 6:**
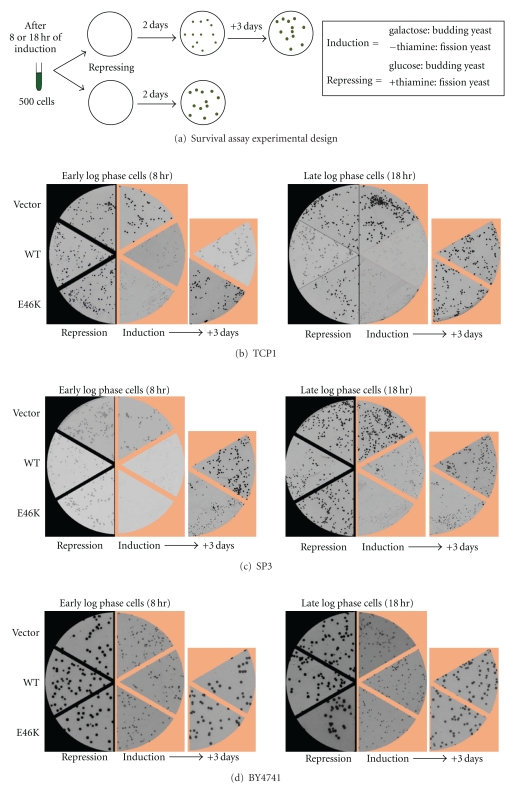
Impaired growth and survival of fission yeast expressing E46K *α*-synuclein. (a) Survival assay schematic: 1000 yeast cells expressing WT or E46K *α*-synuclein or vector alone were first grown in inducing media (without thiamine for fission yeast and galactose for budding yeast) for 8 hours (early log phase) or 18 hours (late log phase) and then plated onto repressing (+thiamine for fission yeast and glucose for budding yeast) or inducing (−thiamine or galactose). Images of plates were taken either two or five days of growth and yeast colonies were examined for size and number (*N* = 3). (b) Composite images of portions of representative plates that contain TCP1 cells grown in repressed conditions for two days or induced conditions for two or five days. (c and d) Same as B, but for SP3 and BY4741 cells, respectively. After two or five total days of continuous expression, E46K *α*-synuclein cells remain significantly smaller than WT in SP3 cells, but not in TCP1 cells or BY4741 cells.

Finally, we asked if the selective E46K *α*-synuclein toxicity in fission yeast was linked to a reduction in organismal growth and survival. As shown in the schematic (Figure 6(a)), 1000 yeast cells each expressing WT or E46K *α*-synuclein or containing parent vector alone for either 8 (early log phase of growth) or 18 hours (late log phase) were plated onto repressing or inducing media. These survival plates were then first grown for two days, and the induced plates were grown even further for three more days. 

Firstly, in the TCP1 strain both E46K and WT *α*-synuclein expressing cells were significantly reduced in colony size compared to parent vector cells when they continued to express *α*-synuclein (*induction=* Figure 6(b)), but not when they stopped expressing *α*-synuclein (*repression*—Figure 6(b)). This was true irrespective of whether *α*-synuclein expressing cells were plated from either early or late log phase of growth. Three days later, both WT and E46K *α*-synuclein cells did gain the original colony sizes initially seen with parent vector cells (Figure 6(b)). We did not observe a reliable difference in colony number between cells that contained parent vector and cells expressing WT or E46K *α*-synuclein (data not shown).

In the SP3 strain, where E46K was shown to be more toxic than WT *α*-synuclein, E46K *α*-synuclein expressing cells formed even smaller colonies than WT *α*-synuclein expressing cells when *α*-synuclein was still being expressed (*induction*—Figure 6(c)). Unlike in TCP1, these E46K colonies did not reach the colony size of parent vector cells even after three days of addition growth even though WT colonies did (Figure 6(c)). Once again, we did not observe a reliable difference in colony number between cells that contained parent vector and expressing WT or E46K *α*-synuclein (data not shown).

In BY4741 budding yeast, similar survival assays did not demonstrate any difference in colony size or number between parent vector, WT, and E46K expressing cells under induced conditions at either two or five days of growth (Figure 6(d) and data not shown). The initial difference in colony size of all yeast cells between repressed (glucose) and induced (galactose) plates was simply due to the slight general lag in yeast growth in galactose compared to glucose.

Thus, the WT and E46K *α*-synuclein toxicity in TCP1 and the additional E46K selective toxicity in SP3 both correlated well with reduced yeast growth but not colony number.

## 3. Discussion

E46K is the most recently discovered of the three *α*-synuclein point mutants that cause familial PD. Since its discovery in 2004, only a handful of mostly *in vitro* studies have evaluated E46K's properties. Using our budding and fission yeast PD models, we demonstrate significant organismal evidence that may help explain its toxic potential in PD, and we discuss three notable findings below. (1) E46K extensively associates with membrane systems in both yeasts but does so in distinct ways: binding plasma membrane in budding yeast and endomembranes in fission yeast. (2) E46K aggregates in fission yeast, but not in budding yeast, in a time- and concentration-dependent manner. (3) E46K is toxic to fission yeast in a strain-specific manner that correlates with reduced cellular growth, survival, and modest accumulation.

### 3.1. E46K Extensively Associates with Membrane Phospholipids

In support of our hypothesis, live cell GFP microscopy illustrates that E46K associates with the plasma membrane of budding yeast. Surprisingly, no plasma membrane association is observed in fission yeast. Instead, in one strain E46K interacts extensively with the cell's endomembrane system. Thus, this study is the first to demonstrate E46K association with diverse membrane compartments in living cells, supporting its well-documented affinity for membrane phospholipids *in vitro*. Since E46K binds liposomes more readily than WT, A30P, or A53T [29], and solution NMR work also suggests that E46K adopts a structure that favors lipid binding more rapidly than these other *α*-synuclein variants [29, 37], these studies provide a molecular explanation for why E46K (more than WT or A53T) strongly associates with endomembrane systems in yeasts. E46K is clearly different from the A30P mutant, which does not associate with membranes within either yeast this study; [45, 46] nor bind lipids *in vitro* [14, 51–53]. Since E46K enhances N- to C-terminal contacts within *α*-synuclein domains [32] and is within the N-terminal KTKEGV amino acid repeats essential for membrane association [10, 30, 54], our data strengthens the notion that the E46K impact on the N to C-terminal contact likely facilitates a helical conformation, the structural form required for *α*-synuclein to interact with lipids [18, 55].

*α*-Synuclein's interactions with membrane systems in cells are well documented. In addition to cytoplasmic localization in neuronal tissue, *α*-synuclein also associates with the secretory pathway [56, 57], localizes to the plasma membrane [16, 58–62], and outside cells [61–65]. Thus, E46K's association (and to a lesser degree, WT and A53T) with internal membrane systems in fission yeast cells is noteworthy. The fission yeast's cortical ER surrounds the nucleus [66]. Thus, E46K *α*-synuclein's prominent presence in perinuclear compartments suggests that it may associate with ER and disrupt ER to Golgi transport to generate toxicity, just as WT and A53T *α*-synuclein have been reported to do in budding yeast cells [26, 27] In budding yeast, overexpression of the secretory pathway specific G protein Rab1 also rescues yeasts from *α*-synuclein-dependent [26, 27].

While we do not know why E46K (and to a lesser extent, WT and A53T) binds such distinct membrane compartments between the two yeasts, differences in *α*-synuclein concentration in the two yeasts are unlikely to be a major reason, since E46K never associates significantly with the fission yeast plasma membrane even when expressed at low concentrations. Since the two yeast species are separated by almost 400 million years of evolution, inevitable differences in plasma membrane and endomembrane composition are a more likely explanation [67]. We speculate that differences in the plasma and endomembrane composition exist between budding yeast and fission yeast and that E46K *α*-synuclein might have increased affinity for plasma membrane phospholipids in budding yeast and endomembranes in fission yeast. Budding and fission yeasts do have modest differences: in phospholipid composition [68], in fission yeast being an inositol auxotroph [69], and fission yeast's lipid composition varies with temperature and growth [70]. Specific differences between the yeasts may also exist in the composition of membrane proteins with which *α*-synuclein variants interact.

### 3.2. E46K Aggregates in Fission Yeast

E46K aggregates more rapidly than WT *α*-synuclein in fission yeast, again supporting our hypothesis. That this, E46K aggregation more closely matches A53T *α*-synuclein is supported by *in vitro* evidence that E46K and A53T aggregate at similar rates [29, 30] In cell culture, E46K readily forms intracellular aggregates [31]. Our work supports the notion that E46K enhances *α*-synuclein aggregation due to its location within the N-terminal KTKEGV repeats, which influence aggregation rates [71]. Synthetic E(XX)K mutations at these points decreased fibrillization lag time *in vitro* [71]. Rospigliosi et al. also demonstrated that the E46K mutant resulted in N to NAC region contact, which, compounded by an overall decrease in negative charge, is hypothesized to increase aggregation rates, providing an explanation for E46K's impact on aggregation [32].

The absence of E46K aggregation in budding yeast may be due to two factors: the moderate expression of *α*-synuclein in our lab and the membrane composition differences between the two yeasts. In budding and fission yeasts, aggregation of *α*-synuclein increases as its concentration is increased [21, 46]. Other labs that express *α*-synuclein at moderate levels also report plasma membrane association [42, 44].

### 3.3. E46K Is Selectively Toxic

E46K's enhanced toxicity in fission yeast supports our hypothesis that it is more toxic than WT *α*-synuclein even though neither of them is toxic in budding yeast. The lack of *α*-synuclein toxicity in budding yeast strain BY4741 could be due to dosage-dependent and strain-specific toxicity, as *α*-synuclein is toxic in the W303 strain with two copies expressed but not with one copy [21]. Our fission yeast data indicates that endomembrane association is a key contributor to toxicity although we do not exclude toxicity contributions from interactions with the plasma membrane or cytoplasmic aggregation. One of the most important questions in PD is the identification of the toxic *α*-synuclein species, and protofibrils possess several features *in vitro* that make them excellent candidates for being this toxic species, including the ability to permeabilize synthetic vesicles [72]. Both A30P and A53T form more protofibrils than WT *α*-synuclein *in vitro* [4, 11, 73], but little still is known whether E46K does the same, *in vitro* or in organisms. We speculate that the differential toxicity (even within fission yeast strains) may be due to how much E46K *α*-synuclein protofibrils form within each strain and that endomembrane association may be a significant contributor.

The degree and range of E46K association with endomembrane systems may determine how much it contributes to cytotoxicity. Even though E46K localizes more to internal organelles in TCP1 than WT *α*-synuclein does, their level of toxicity is similar. We speculate that the added toxicity in SP3 may partly be due to more E46K localization to vacuoles/prevacuoles. Thus, disruption of vesicle integrity may mediate toxicity as much as added association with the ER. Strain differences in lipid composition may also underlie why SP3 differs from TCP1 in the extent of *α*-synuclein association with intracellular organelles. Since most TCP1 cells express E46K in aggregates, the few cells that do exhibit endomembrane localization likely produce too little toxicity to be detected by the growth and survival assays. Lastly, the difference in toxicity could be due to the h− (TCP1) and h+ (SP3) mating-type differences, including altered interactions of *α*-synuclein with secretion of specific mating factors. 

Our data confirms an initial report in budding yeast [74] and extends it to fission yeast that yeasts possess protein kinases that can phosphorylate human *α*-synuclein. We are also the first to report that WT and E46K *α*-synuclein are phosphorylated in a fission yeast model (for fuller description, see [75]). Although the extent of serine-129 phosphorylation does not correlate with enhanced E46K *α*-synuclein toxicity in fission yeast, it provided us the opportunity to investigate the contribution of phosphorylation to *α*-synuclein aggregation and membrane association in both models that we report elsewhere.

Future studies in fission yeast will determine whether *α*-synuclein will disrupt vesicular pool distribution, accumulates in vesicles, and colocalizes with secretory pathway proteins, as it does in budding yeast [21, 27, 76]. It is possible that such *α*-synuclein endomembrane association also impairs *α*-synuclein degradation and toxicity via lysosmal pathways, as has been observed in budding yeast ([47, 77], Perez et al. (manuscript submitted), Konnikova et al. (manuscript in prep)), mammalian cells [78–82] and worms [83].

## 4. Conclusion

Both yeast models recapitulate and extend our *in vitro *and cell culture knowledge of E46K *α*-synuclein familial mutant properties in two organismal model systems. Together, they illustrate that E46K can both aggregate within cells and associate with its diverse membrane systems. Importantly, the organismal context regulates both these features by varying the extent of aggregation and the extent of association with plasma membrane and endomembrane systems. Our data indicates that *α*-synuclein binding to endomembranes may be key to generating toxicity that negatively impacts cellular growth and survival. Finally, our work further strengthens the relevance of fission yeast, alongside the more widely accepted budding yeast, to serve as a model organism for studying misfolded proteins linked to human neurodegeneration, opening doors for genetic screens and chemical treatments to dissect the role of genetic factors that regulate *α*-synuclein misfolding and toxicity and contribute to deeper understanding of PD pathogenesis.

## 5. Materials and Methods

### 5.1. *α*-Synuclein Constructs

cDNAs of wild-type (WT) and A53T *α*-synuclein were provided by Christopher Ross at Johns Hopkins University. These *α*-synuclein cDNAs were first subcloned into the mammalian expression vector pcDNA3.1/C-terminal GFP (Invitrogen), fusing *α*-synuclein with GFP at the C-terminus. The *α*-Synuclein-GFP cDNAs and GFP cDNA were then PCR-amplified and subcloned into the pYES2.1/V5-His-TOPO budding yeast expression vector (Invitrogen) or pNMT1/V5-His-TOPO fission yeast vector (Invitrogen). The *α*-synuclein-GFP fusion yeast vectors were transformed into chemically competent *E*.* coli* cells for replication and storage. The A30P mutant was created using site-directed mutagenesis (Invitrogen) on WT *α*-synuclein-GFP pYES2.1/V5-His-TOPO budding yeast expression vector (Invitrogen) or pNMT1/V5-His-TOPO as described in Sharma et al. and Brandis et al., respectively [45, 46]. The E46K mutant was made similarly created with the following primers:

E46K FP: 5′ATGTAGGCTCCAAAAACAAGAAGGGAGTGGTGC 3′
E46K RP: 5′CTTGGTTTTGGAGCCTACATAGAGAACACC 3′

Mutations were sequenced at University of Chicago to confirm successful substitution. The parent pYES2.1 vector for budding yeast (Invitrogen) and parent pNMT1 pREP vector (gift from Judy Potashkin, Rosalind Franklin University, Ill, USA) served as controls.

### 5.2. Yeast Strains

Parent budding yeast strains BY4741 (MATa *his3Δ1 leu2Δ0 met15Δ0 ura3Δ0*), BY4742 (MAT*αhis3Δ1 leu2Δ0 lys2Δ0 met15Δ0 ura3Δ0*), and BY4743 (MATa/*α his3Δ1 leu2Δ0 lys2Δ0 ura3Δ0*). Fission yeast strains TCP1 (h− *leu1-32)* and SP3 (h+ *leu1-32*) were provided by Invitrogen and Judy Potashkin (Rosalind Franklin University of Medicine and Science, North Chicago, Ill, USA), respectively.

### 5.3. *S. cerevisiaeα*-Synuclein Expression

*α*-Synuclein expression plasmid vectors were transformed into yeast strains as described [84]. For selection, yeast cells were grown on synthetic-complete media lacking uracil (SC-Ura). In the pYES2 vector, *α*-synuclein expression was controlled through a tightly regulated galactose-inducible promoter (Gal1). Yeast were grown to mid-log phase in SC-Ura glucose (2%) or SC-Ura raffinose (2%) media at 30°C. Cells were washed with water and diluted to log-phase (5 × 10^6^ cells/mL) in SC-Ura galactose (2%) media to induce *α*-synuclein expression.

### 5.4. *S. pombeα*-Synuclein Expression

*S*.* pombe* strains were transformed with pNMT1 vectors using the lithium-acetate transformation method [85]. Transformed cells were selected by growth on *pombe* dropout medium-leucine (PDM-Leu) containing 10 *μ*M thiamine. *α*-Synuclein was expressed by growth in thiamine-lacking media as described in [46].

### 5.5. Western Analysis

Budding or fission yeast cells at 2.5 × 10^7^ cells/mL concentration were washed twice with 100 mM NaN_3_ and solubilized in electrophoresis sample buffer (ESB) [84]. The ESB contained 2% sodium dodecyl sulfate (SDS), 80 mM Tris (Ph 6.8), 10% glycerol, 1.5% dithiothreitol, 1 mg/mL bromophenol blue, and a cocktail of protease inhibitors and solubilizing agents (1% Triton-X 100, 1 mM phenylmethylsulfonyl fluoride, 1 mM benzamidine, 1 mM sodium orthovanadate, 0.7 mg/mL pepstatin A, 0.5 mg/mL leupeptin, 10 mg/mL E64, 2 mg/mL aprotinin, and 2 mg/mL chymostatin). Lysates were electrophoresed at 130 volts on a 10%–20% Tris-Glycine gel (Invitrogen) with 1X SDS running buffer. SeeBlue (Invitrogen) molecular ladder was used as a standard. Gels were transferred to PVDF membranes using a semidry transfer method and probed using the desired antibodies. To detect *α*-synuclein, a mouse monoclonal anti-V5-AP antibody (Invitrogen) was used at 1 : 2000. Mouse antiphosphoglycerokinase (PGK; Molecular probes) was used at 1 : 1000 as a loading control for budding yeast and anti-*β*-actin (Abcam) was used at 1 : 1000 as a loading control for fission yeast. For both, goat antimouse secondary antibody (Invitrogen) was used. Serine-129 phosphorylation blots were probed with a rabbit *α*-synuclein (phospho S129) antibody (ab59264; Abcam) at 1 : 500, followed by a goat antirabbit secondary antibody (Santa Cruz Biotechnology). All blots were visualized by detecting for alkaline-phosphatase activity. All blots were done at least three times.

### 5.6. OD600 Growth Curve Analysis

Yeast cells were grown in either 10 mL SC-Ura+glucose (budding yeast) or EMM+T (fission yeast) overnight at 30°C and 200 rpm. To collect cells, yeast were pelleted at 1500 × g for 5 minutes at 4°C. They were washed twice with 5 mL H_2_O, re-suspended in 10 mL H_2_O, and counted using a hemocytometer to determine cell density. Flasks containing 25 mL SC-Ura+galactose (budding yeast) or EMM−T (fission yeast) were inoculated to a density of 2.0 × 10^6^ cells/mL. Duplicate spectrophotometer 600 nm absorbance measurements of 1 mL of cells in a plastic cuvette were taken at 0, 3, 6, 12, 18, 24, 36, and 48 hours after-induction. The spectrophotometer model was a Hitachi U-2000 Spectrophotometer. A growth curve was generated by plotting the average absorbance readings of three separate experiments versus time in Microsoft Excel. A student's *t*-test was used to determine significance.

### 5.7. Serial Dilution Spotting

Yeast cells were grown in either 10 mL SC-Ura+glucose (budding yeast) or EMM+T (fission yeast) overnight at 30°C and 200 rpm. To collect cells, yeast were pelleted at 1500 × g for 5 minutes at 4°C. They were washed twice with 5 mL H_2_O, resuspended in 10 mL H_2_O, and counted using a hemocytometer to determine cell density. 2.0 × 10^6^ cells were removed and pelleted. The supernatant was removed, and cells were resuspended in 1 mL H_2_O. Cells were diluted 5 fold in a 96 well microtitier plate and spotted onto SC-Ura+glucose and Sc-Ura+galactose (budding yeast) or EMM+T and EMM−T (fission yeast) growth plates. Cells were grown for 24 hours, and pictures were taken using an HP Canoscan scanner. Images were imported into Adobe Photoshop CS2. All spotting experiments were conducted at least three times in triplicate.

### 5.8. GFP Microscopy

Yeast cells were grown in either 10 mL SC-Ura+glucose (budding yeast) or EMM+T (fission yeast) overnight at 30°C and 200 rpm. To collect cells, yeast were pelleted at 1500 × g for 5 minutes at 4°C. They were washed twice with 5 mL H_2_O, resuspended in 10 mL H_2_O, and counted using a hemocytometer to determine cell density. Flasks containing 25 mL SC-Ura+galactose (budding yeast) or EMM−T (fission yeast) were inoculated to a density of 2.0 × 10^7^ cells/mL. 1 mL of cell culture was pelleted at 5000 rpm for 1 minute. 900 uL of supernatant was removed. The remaining 100 uL of cell culture was vortexed, and 5–10 uL of sample was pipetted onto a glass slide. Cells were visualized using a Nikon TE2000-U fluorescent microscope, and images were collected and quantified using Metamorph 4.0 software. All microscopy experiments were conducted twice. For each yeast sample, at each time point ~750 cells of each type were scored for all or some of these localization patterns (depending on the yeast): cytoplasmically diffuse, plasma membrane, aggregate, membrane/diffuse, membrane/aggregate, endomembrane, endomembrane/aggregate, and endomembrane/diffuse (*bottom*). All microscopy experiments were conducted twice. DAPI staining was performed as described [85].

### 5.9. Survival Assay

Yeast cells were grown in either 10 mL SC-Ura+glucose (budding yeast) or EMM+T (fission yeast) overnight at 30°C and 200 rpm. To collect cells, yeast were pelleted at 1500 × g for 5 minutes at 4°C. They were washed three times with 5 mL H_2_O, resuspended in 10 mL H_2_O, and counted using a hemocytometer to determine cell density. Flasks containing 25 mL SC-Ura+galactose (budding yeast) or EMM−T (fission yeast) were inoculated to a density of 2.0 × 10^7^ cells. At 8 and 18 hours after induction, cell density was determined. 1.8 × 10^6^ cells were removed and washed once with 1 mL of H_2_O. The cells were resuspended in 1 mL of water, and diluted 1 : 1000. 300 *μ*L of 1 : 1000 diluted cells (550 cells) were spread on 150 mm plates containing SC-Ura+Galactose and SC-Ura+Glucose (budding yeast) or EMM−T and EMM+T (fission yeast), for either two days or three additional days at 30°C, and images were taken of plates and analyzed for colony number and size. All survival assays were conducted at least three times in triplicate.
